# Farmers’ Risk Cognition, Risk Preferences and Climate Change Adaptive Behavior: A Structural Equation Modeling Approach

**DOI:** 10.3390/ijerph17010085

**Published:** 2019-12-20

**Authors:** Rui He, Jianjun Jin, Foyuan Kuang, Chenyang Zhang, Tong Guan

**Affiliations:** 1 State Key Laboratory of Earth Surface Processes and Resource Ecology, Beijing Normal University, Beijing 100875, China; 201621190023@mail.bnu.edu.cn; 2 Faculty of Geographical Sciences, Beijing Normal University, Beijing 100875, China; hzgong@163.com (F.K.); xuxia02@126.com (C.Z.); chunyanghe09@163.com (T.G.); 3 Chongqing Academy of Social Sciences, Chongqing 40020, China

**Keywords:** climate change adaptive behavior, risk cognition, risk preference, structural equations, rural China

## Abstract

Improving local farmers′ climate change adaptive capacity is an important policy issue in rural China. This study investigates farmers′ risk cognition, risk preferences and climate change adaptive behavior. Based on unique data from a survey and a paired lottery experiment completed by 240 rural farmers in Chongqing City of China, this paper finds that farmers have a pessimistic risk cognition towards climate change and the typical farmers are risk-averse and loss-averse. Risk cognition and adaptation cognition have significantly positive influences on climate change adaptive behavior, and loss aversion has a significantly positive influence on farmers′ adaptation decisions. Loss aversion exerts a positive impact on risk cognition and adaptation cognition, and risk aversion has a positive impact on adaptation cognition. This paper contributes to the emerging literature that relates risk preference in experiments and risk cognition to farmers′ climate change adaptive behavior.

## 1. Introduction

Climate change has a great impact on agriculture [1,2]. Adaptation is a policy option to mitigate the adverse effects of climate change [3]. Only by adopting reasonable adaptation measures can farmers effectively mitigate the possible negative impacts of climate change [4]. In coping with climate change, as the main body of agricultural productions, there is an urgent need to improve farmers′ adaptive capacity [5,6,7].

Pratt [8] and Arrow [9] argue that risk cognition and risk preference are two main factors influencing individual decision-making under the condition of risk and uncertainty. Risk cognition is an individual’s perception of risk. Public perceptions of risk guide their behaviors largely, and the cognition of climate change is the basis for farmers to adopt adaptive behavior [10,11]. Li et al. [12] find that the awareness of extreme weather event risks was a significant driver of adaptation behavior. Mase et al. [13] highlight the role of risk perception in farmers’ climate change adaptation strategies. Azadi et al. [14] report that human cognition is an important determinant of climate change adaptation. Risk preference is the attitude of a decision maker to risks. It is another important factor for individuals to perceive decision-making background and make decisions towards risk [15,16,17,18]. Tanaka et al. [19] propose that individuals’ risk preference includes risk aversion and loss aversion. The term risk averse refers to investors who, when faced with two investments with a similar expected return, prefer the lower-risk option. Loss aversion is based on Prospect Theory which argues that individuals behave differently in the domain of gain and in the loss domain. People may find the losses more unbearable when facing the same amount of gains and losses. Bartczak et al. [20] explore how risk preference and loss aversion affect individuals’ choices of environmental risk, especially in Polish forest fires. Tong et al. [21] find most farmers are risk-averse and this risk aversion makes farmers less efficient in the use of climate risk management inputs. Jin et al. [22] point out that farmers**′** risk aversion was negatively and significantly related with adaptation strategies on adopting new technology, and it has a significantly positive effect on buying weather index crop insurance. Thus, when facing the impact of climate change, farmers may take adaptation measures based on the possibility of risk and loss occurrence.

In view of risk cognition and actual behavior, Pennings et al. [23] thought that risk cognition can be used to analyze individual behavior effectively only when it is combined with risk preference. For the relationship between risk cognition and risk preference, there are different opinions in the literature. There is a mediating effect hypothesis [24] and a moderating effect hypothesis [25]. In fact, most of the studies only use econometric regression methods to study the impact of risk cognition or risk preferences on farmers’ adaptation behavior. However, econometric regression methods cannot show the indirect and direct interrelationships that may exist among multiple variables [26]. The structural equation model (SEM) is a relatively new and comprehensive method that can deal with the relationship between multiple causes and results [27]. It is a special form of multivariate analysis that examines the roles and inter-relationships of multiple variables, which can be used to determine the relationship among farmers’ risk cognition, risk preferences and climate change adaptive behavior. To our best knowledge, the study dealing with farmers’ climate change adaptive behavior by using the SEM is very limited in China.

Based on the research progress mentioned above, this paper aims to investigate farmers’ climate change adaptive behavior based on risk cognition and risk preference by using a structural equation modeling approach. This study may contribute to the existing literature in the following aspects: (1) farmers’ climate change adaptive behavior were identified and investigated based on local natural, institutional and socioeconomic factors; (2) farmers’ risk cognition was measured based on the Private Proactive Adaptation to Climate Change model; (3) economic experiments were used to measure farmers’ risk preference and loss aversion; (4) the structural equation model was employed to analyze the specific relationships among farmers’ risk preference, risk cognition and climate change adaptive behavior. The results from this paper can be used by other researchers as well as policy-makers for promoting policies of climate change adaptation in China and even other developing countries.

This paper first provides the methods that include the study area, the survey design, the experimental design, the structural equation model, the sample and data collection. These sections are followed by the empirical results and discussions. Then a summary of the findings follows.

## 2. Methods

### 2.1. Study Area

This study chose Dazu district in the west of Chongqing municipality as the study area. Chongqing municipality is one of the four municipalities directly controlled by the central government of China. The total GDP of Dazu district was 5.73 billion USD in 2016 [28]. The total agricultural population of Dazu district was 0.58 million, covering 55.17% of the total population. Dazu is the national commodity grain production county and agricultural production is very important in this area. However, the area has been affected by climate change, and the disastrous climate has had a great influence on agricultural productions and farmers’ livelihoods. Thus, it is very vital in understanding farmers’ perceptions of and responses to climate change.

### 2.2. Survey Design

A questionnaire survey was conducted in the study area. The questionnaire was developed on the basis of a series of focus group discussions and a preliminary survey. The research team invited some experts in risk experiments, local government officials in agriculture and local farmers to participate in the discussions. The purpose was to collect information that is specific on local farmers’ climate change adaptive behavior and to solicit their opinions on the research design. The original questionnaire was modified based on the pilot survey results.

The final questionnaire used in the survey consisted of three main sections. The first section contained questions about farmers’ climate change adaptive behavior. Each respondent was asked, in order to cope with climate change, whether they have planted new crop varieties, adjusted pesticide application, adjusted fertilizer application, improved irrigation methods/frequency, diversified planting or not. The second section investigated a wide range of farmers’ risk cognition. Several statements such as “climate change has severely affected your daily life”, “the possibility of climate change in the future will be very high”, “taking adaptation actions can mitigate the effects of climate change”, “my ability to deal with climate change is very high” and “the cost of climate change adaptation measures is low” were presented where respondents were asked to express the extent of their agreement on a five-point Likert scale rating from “strongly disagree” to “strongly agree”. These five questions corresponded to five kinds of cognition that were called severity cognition, possibility cognition, adaptation efficacy cognition, self-efficacy cognition and adaptation cost cognition. Moreover, the first two perceptions are associated with climate change risk cognition, and the last three perceptions are related to adaptation cognition [29]. The last section of the survey collected socio-economic characteristics of the respondents and their households (e.g., gender, age, level of education, number of labors, number of family members and household income). The main structure of the survey is shown in the Appendix A.

### 2.3. Experimental Design

To elicit farmers’ risk preference and loss aversion, our research team designed a field experiment referring to the research of Liu et al. [30]. This design is among the most commonly used experimental methods to elicit individuals’ risk preference [20]. The experiment included a series of choices to measure farmers’ risk aversion and another series to elicit farmers’ loss aversion.

#### 2.3.1. Risk-Aversion Experiment

The risk-aversion experiment was based on Holt and Laury [31] and Brick et al. [32], which has been widely used in the developing countries, showing that even low-educated farmers can understand the design [30].

Table 1 shows the eight binary-choice questions where farmers should make their choices between a safe option and a risky option. From task 1 to task 8, the token of the safe option (option A) is gradually reduced from 200 to 150, 120, 100, 80, 60, 40, 20 and the probability of winning is 100%. As for the risky option (option B), the farmers may gain 200 with a 50% probability or gain 0 with a 50% chance in any task. It can be seen that the higher token in option B (200) is always higher than the token in option A, except for the first task. However, there is a 50% chance in option B gaining zero payment. In fact, payoffs of the safe option have been declining gradually, while payoffs of the risky option stay the same all the time. EV_A_-EV_B_ in Table 1 shows the difference of the expected values of option A and option B. It can be seen that EV_A_-EV_B_ is positive from task1 to task3 and equals to zero in task 4, then is less than zero from task 5 to task 8. Based on this, a risk-seeking farmer will choose the safe option less than three times, while a risk-averse farmer may choose the safe option more than four times. Moreover, a risk-neutral farmer will pick the safe option (option A) for the first three or four tasks and then switch to the risky option (option B).

#### 2.3.2. Loss-Aversion Experiment

To obtain farmers’ attitudes toward loss, this study used the loss-aversion experiment design based on Liu at al. [33]. Farmers would make a choice between option A and option B from task 1 to task 7 (Table 2). As for each task, option A (the safe option) and option B (the risky option) both have a 50% possibility of gaining tokens and a 50% possibility of losing tokens. The possible tokens in option B (gaining 75 tokens) are always higher than that in option A (gaining 35–60 tokens), while the possible loss amount in option B (losing 40–65 tokens) is always lower than that in option A (losing 35 tokens). In other words, option B has better gains and also higher losses than option A. EV_A_-EV_B_ in Table 2 also shows the difference of the expected values of option A and option B. From task 1 to task 3, the expected payoff of option A is higher than the expected payoff of option B, and the expected payoff of option A and option B is the same in task 4 which equals zero. At the same time, the expected payoff of option A is lower than the expected payoff of option B from task 4 to task 7 and the value of EV_A_-EV_B_ is less than zero. Theoretically, a risk-neutral and not loss-averse farmer may choose option A for the first three tasks, and will be indifferent between options A and B in the fourth task, and will choose option B thereafter. In addition, a risk-neutral and loss-averse farmer will not be indifferent between option A and option B in Task 4 due to option B has more loss than option A.

### 2.4. Sample and Data Collection

The research team conducted the field survey and risk experiments in July 2017. Respondents were randomly selected. First, the research team randomly selected two towns, Zhongao and Sanqu, according to their size and population. Then, three villages were randomly selected in each town. According to the population size of each village, the research team randomly selected 30−50 households for the survey and experiment. Trained investigators conducted face-to-face interviews with farmers. Experiments to measure farmers’ risk preference were run after the questionnaire survey. To ensure the validity of the risk experiment, participants were informed that they would receive cash payments after the experiment. This payment is closely related to their experimental choices and they must make their decisions in the experiment seriously.

Finally, each participant received $1.50 for completing the questionnaire survey and an actual cash payment from the risk experiment. A total of 240 responses were eventually collected, of which 228 were found to be valid for further analysis.

### 2.5. The Structural Model

In order to clarify the relationships between farmers’ risk cognition, risk preference and climate change adaptive decisions, the SEM was employed. Since risk aversion and loss aversion are two aspects of risk preference, the risk-aversion coefficient and loss aversion coefficient enter the model as observation variables. At the same time, in the model fitting, residual variable was introduced to give the model higher moderate error, and the default path coefficient of the model is 1. In this paper, Amos 21.0 from IBM company was used as a tool to obtain SEM results.

This paper proposes the hypothesis that risk aversion has a significant and positive impact on climate change adaptive behavior. In addition, farmers who are more loss-averse may be more willing to take actions against climate change based on the cognition that they are taking steps to reduce losses. At the same time, farmers’ cognition of climate change risks has a positive impact on their climate change adaptive behavior. Based on the definition of adaptation cognition in this paper, it is believed that adaptation cognition has a significant and positive influence on climate change adaptive behavior; that is to say, people with higher adaptation cognition are more likely to adopt climate change adaptive behavior. In addition, this paper proposes that the more risk-averse and loss-averse farmers are, the more likely they are to have positive risk cognition, and the more likely they are to perceive the severity and possibility of risk. With positive adaptation cognition, they are more willing to take countermeasures to cope with climate change.

**Hypothesis** **1** **(H1)**. *Risk aversion has a significant and positive impact on climate change adaptive behavior*.

**Hypothesis** **2** **(H2)**. *Loss aversion has a significant and positive impact on climate change adaptive behavior*.

**Hypothesis** **3** **(H3)**. *Risk cognition has a significant and positive impact on climate change adaptive behavior*.

**Hypothesis** **4** **(H4)**. *Adaptation cognition has a significant and positive influence on climate change adaptive behavior*.

**Hypothesis** **5** **(H5)** *Risk aversion has a significant and positive effect on risk cognition.*

**Hypothesis** **6** **(H6)** *Risk aversion has a significant and positive effect on adaptation cognition.*

**Hypothesis** **7** **(H7)** *Loss aversion has a significant and positive effect on risk cognition.*

**Hypothesis** **8** **(H8)** *Loss aversion has a significant and positive effect on adaptation cognition.*

## 3. Results and Discussion

### 3.1. Participants

The descriptive socio-economic characteristics of the respondents is reported in Table 3. Approximately, male farmers represented 49% of our sample. The typical respondent was 59 years old, ranging from 20 to 80 years. One reason for the absence of young respondents is that many young farmers leave villages to work in big cities for better jobs and payments. In addition, the average household had three labors. On average, respondents had attained about six years of education (primary school), indicating the educational level of our respondents was low. The overall average household size of the sampled respondents was about five persons. Each household owned about 0.22 hectare of farmland. The average household income was approximately 610 USD/month. Our sample is representative, comparing with the data from Chongqing Statistical Yearbook 2017 (recording the data of 2016) [28].

### 3.2. Farmers’ Risk Cognition

Based on the social cognitive model developed by Grothmann and Patt [29], this paper investigated five kinds of climate change cognition of farmers, namely, severity cognition, possibility cognition, adaptation efficiency cognition, self-efficacy cognition and the adaptation cost cognition. The results are shown in Table 4.

As can be seen from Table 4, 68.86% of farmers agreed or strongly agreed that climate change had seriously affected their lives. There were 62.28% of respondents who agreed or strongly agreed that it is very likely to have further climate change in the future. Approximately, 42.54% of farmers were not sure that taking adaptation measures can mitigate the adverse effects of climate change. The results also reveal that 41.67% of the farmers thought their ability to cope with climate change was low. Approximately 75.88% of the farmers thought that the cost of taking counter adaptation measures was high.

### 3.3. Farmers’ Risk Preference

Table 5 shows the descriptive statistics of farmers’ switching point. There is a certain percentage of cross choices in the risk-aversion experiment and loss-aversion experiment. Moreover, the results show that only 6.58% shows risk-loving behavior in the risk-aversion experiment. Approximately 14.03% of farmers exhibited risk-neutral behavior. Most farmers (79.39%) made risk-averse choices, while in the loss-aversion experiment, 15.35% of farmers exhibited a low degree of risk aversion. Around 13.16% of farmers were loss-neutral, while approximately 66.67% of farmers showed loss-averse preferences. 

In addition, this paper further uses the utility function to calculate the risk-aversion coefficient and loss-aversion coefficient. The mean of the risk-aversion coefficient was 0.22 and the standard deviation was 0.67. This result is consistent with Tanaka et al. [19], Harrison et al. [34], Brick et al. [32] and Liu et al. [30,33]. The mean value of the loss-aversion coefficient was 2.87 (standard deviation: 2.63), which is close to the results of 2.25 in the literature of Tversky and Kahneman [35] and 2.63 in the paper of Tanaka et al. [19]. This finding suggests that most farmers in the study area are loss-averse. All the above results show that the majority of farmers in the study area are risk-averse and loss-averse.

### 3.4. Farmers’ Climate Change Adaptive Behavior

The survey results show that most farmers have adopted a variety of adaptation measures to cope with climate change, and only 14 farmers did not take any adaptation actions. The specific adaptation measures taken by local farmers are shown in Table 6. The results show that 72.81% of the farmers had planted new tolerant crop varieties. Approximately 64.91% of the farmers had adjusted their pesticide use behavior to cope with climate change, and 59.65% of the farmers had adjusted the application of fertilizer. The results also show that 42.54% of the farmers had adjusted irrigation methods/frequency to deal with climate change. The rate of the farmers who chose diversified planting was 38.16%.

### 3.5. The Overall Relationships among Farmers’ Risk Preference, Risk Cognition and Climate Change Adaptive Behavior

**Figure 1:**
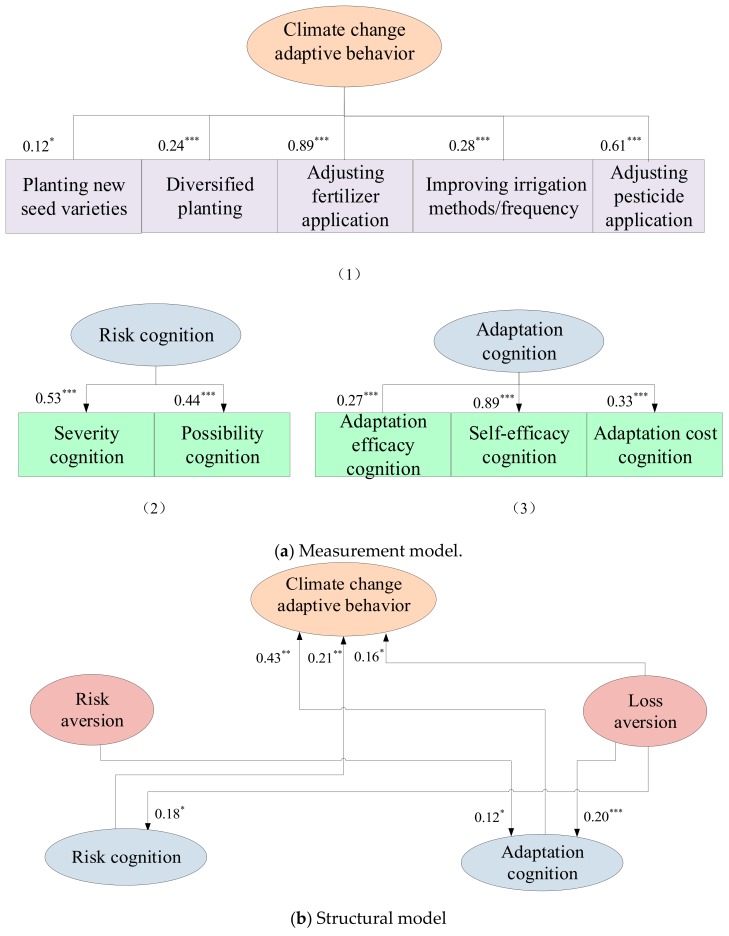
The final structural equation model.

This study constructs the structural equation model according to the hypothesis and variable setting. The results show that the latent variables of climate change adaptive behavior, and farmers’ cognition of risk and adaptation in the conceptual model all passed the significance test. The goodness-of-fit statistics indicated the reported model was acceptable (statistical indicators of the model: *χ^2^* = 57.63, *p* = 0.06, *χ^2^*/*df* = 1.37; goodness-of-fit index (GFI) = 0.96; root mean square residual (RMR) = 0.04; root mean square error of approximation (RMSEA) = 0.04; incremental fit index (IFI) = 0.95; Tacker-Lewis index (TLI) = 0.91; comparative fit index (CFI) = 0.94; parsimony-adjusted CFI (PCFI) = 0.60; parsimony-adjusted normed fit index (PNFI) = 0.53). The evaluation indexes of the model’s overall fitting degree all passed the test, and the overall path significance of the model was significant. Therefore, the analysis results are reliable and shown in Figure 1.

#### 3.5.1. The Internal Relationship of Farmers’ Risk Preference, Risk Cognition and Climate Change Adaptive Behavior

The estimated results in the measurement model of standardized parameters of paths between observation variables and latent variables are all significant, indicating that each indicator has a strong explanatory ability. Among the three latent variables, the latent variable of climate change adaptive behavior consisted of five indicators, i.e., planting new seed varieties, diversified planting, adjusting of fertilization behavior, improving irrigation method/frequency, and adjusting pesticide application behavior. The factor relationship values of the five factors were 0.12, 0.24, 0.89, 0.28, and 0.61, respectively. The explanation ability of adjusting fertilization use behavior for the latent variable was the strongest, followed by adjusting pesticide application behavior, the improvement of irrigation method/frequency, diversified planting and finally planting new seed varieties.

The latent variable of risk cognition is composed of two indicators: possibility cognition and severity cognition. According to the standardized estimation results of the two paths, the factor relationship between possibility cognition and risk cognition was 0.44, and the factor relationship between severity cognition and risk cognition was 0.53. This means that the interpretation power of the possibility cognition was a little weaker than the severity perception. The signs of these two cognition variables were positive. This implies that when farmers perceive the adverse impacts of climate change and consider climate change to affect their lives, they are more likely to take adaptation measures to cope with climate change [29].

The latent variable of adaptation cognition consists of three indicators: adaptive efficacy cognition, self-efficacy cognition and adaption cost cognition. The factor relationship coefficients of the three factors for adaptation cognition were 0.27, 0.89 and 0.33, respectively, which means that self-efficacy cognition has the strongest explanation power, followed by the adaptation cost cognition and the self-efficacy cognition. This finding is in line with previous research. Farmers would take adaptation actions only when they feel capable of coping with the risk [36]. Uncertainties regarding the effectiveness of adaptation measures can impede the adaptation efforts. Moreover, adaptation cost is also an important factor influencing farmers’ adaptation decisions. Programs that would lower farmers’ adaptation cost could be launched to improve farmers’ adaptive ability.

#### 3.5.2. The Cross Relationship among Farmers’ Risk Preference, Risk Cognition and Climate Change Adaptive Behavior

The maximum likelihood (ML) estimation method was used to estimate the causal relationship between the potential variables and path coefficients. The results show that risk cognition and adaptation cognition play an intermediary role in the influence of loss aversion on climate change adaptation. This result supports the “mediation hypothesis” [16]. The results also show that risk cognition and adaptation cognition have significant and positive influences on climate change adaptive behavior. This finding is consistent with the literature [37,38,39,40]. Farmers who have better knowledge on climate change risks and adaptation are more likely to take adaptation actions. Combined with the definitions of variables, the results indicate that farmers who believed that climate change had a serious impact on their lives and that there was a high possibility of future climate change are more willing to take measures to deal with climate change. This is understandable because farmers who have perceived negative impacts of climate change and who judged the risk of climate change to be high were more likely to adopt adaptation measures. At the same time, farmers who believed that taking measures can mitigate the negative impacts of climate change, that they had a high capacity to cope with climate change, and that the cost of taking measures was relatively low were more willing to take measures to cope with climate change. This result is consistent with earlier findings. Truelove et al. [38] indicate that behavior-specific efficacy beliefs and capability beliefs are important predictors for adopting agricultural adaptations. This study also proves that the risk cognition and adaptation cognition should be studied separately as two dimensions following the study of Grothmann and Patt [29].

The results of the structural equation model show that loss aversion has a significant and positive influence on climate change adaptive behavior. This suggests that farmers with higher loss aversion are more willing to adopt climate change adaptation measures. In addition, loss aversion has a significant and positive impact on risk cognition and adaptation cognition. This may be because farmers with a higher degree of loss aversion can perceive the severity and possibility of risks and have a higher cognition of adaptation. Risk aversion has a significant and positive impact on adaptation cognition, indicating farmers who are more risk-averse have higher adaptation cognition. The study did not find that the degree of risk aversion has a significant impact on the adaptive behavior of farmers. This result was not in line with expectations. Further research should be conducted to better understand the role of risk aversion on climate change adaptation behavior.

In the action effect of structural variables, in addition to direct effects between variables, there were indirect effects. Indirect effects may express some complex relationships of actions. In a causal relationship, the sum of direct and indirect effects was the total effects between the starting variable and the ending variable. It can be found that only loss aversion has indirect effects on climate change adaptive behavior in this study. Specifically, except its direct effects on climate change adaptive behavior, loss aversion has an indirect effect on farmers’ climate change adaptive behavior through risk cognition and adaptation cognition. This finding supports the mediating effect hypothesis [24].

## 4. Conclusions

It is becoming important to promote the ability of local farmers to adapt to climate change. A better understanding of farmers’ perceptions of and responses to climate change is important for decision-makers to design more effective adaptation policies. Individuals’ risk preference and loss aversion have been identified as factors affecting farmer’s climate change adaptive behavior. However, there is little empirical evidence of an interactive relationship between farmers’ risk cognition, risk preferences and adaptive behavior. Taking Dazu district of Chongqing as the study area, this paper investigates farmers’ risk cognition, risk preferences and climate change adaptive behavior based on a unique data from a questionnaire survey and a paired lottery experiment completed by 240 randomly selected rural farmers.

The results show that more than half of the respondents believed that climate change had affected their lives and the possibility of climate change in the future was high. Most farmers thought their abilities to cope with climate change were low and the cost of taking adaptation measures was high. Although some farmers were not sure that taking adaptation measures can mitigate the effects of climate change or not, many farmers have adopted certain adaptation measures to cope with climate change. The risk experiment results show that most farmers are risk-averse and loss-averse. The results of the structural equation model reveal that risk cognition and adaptation cognition play a mediating role in the impact of loss aversion and have significant positive effects on the decisions of climate change adaptive behavior. At the same time, risk aversion has a significant and positive impact on adaptation cognition, but has no effect on farmers’ adaptive behavior. Loss aversion has a significant and positive impact on risk cognition, adaptation cognition and climate change adaptive behavior.

The findings of this study provide some policy implications for China and other similar developing countries. Firstly, more adaptive guidance from the government should be given to farmers and some financial subsidies may be considered. Judging from the five-dimensional cognitive results of farmers in this paper, some positive cognitive and psychological guidance is important for farmers. Secondly, attaching importance to the study of loss aversion may have a positive impact on farmers’ risk cognition and adaptation behavior. Although other studies have looked more at the effects of risk aversion on different adaptive behaviors, there has been little research on loss aversion in China. More attention and information should be added to the research of risk preference. Finally, it is necessary to comprehensively consider risk preference and risk cognition on farmers’ adaptation behavior. Focusing only on risk preference or risk cognition on farmers’ behavior may be insufficient and incomplete because of the correlation between risk preference and risk cognition. The dissemination of more correct information to farmers is also necessary because of the mediating role of cognition in the study.

## Figures and Tables

**Table 1:** Risk-aversion experiment design.

Task	Option A		Option B				EV_A_-EV_B_
	Tokens	Prob.	Tokens (1)	Prob.	Tokens (2)	Prob.	
1	200	100%	200	50%	0	50%	100
2	150	100%	200	50%	0	50%	50
3	120	100%	200	50%	0	50%	20
4	100	100%	200	50%	0	50%	0
5	80	100%	200	50%	0	50%	−20
6	60	100%	200	50%	0	50%	−40
7	40	100%	200	50%	0	50%	−60
8	20	100%	200	50%	0	50%	−80

Note: EV_A_ and EV_B_ are the expected value of option A and option B, respectively.

**Table 2:** Loss-aversion experiment design.

Task	Option A				Option B				EV_A_-EV_B_
	Tokens (1)	Prob.	Tokens (2)	Prob.	Tokens (1)	Prob.	Tokens (2)	Prob.	
1	60	50%	−35	50%	75	50%	−65	50%	7.5
2	55	50%	−35	50%	75	50%	−65	50%	5
3	50	50%	−35	50%	75	50%	−65	50%	2.5
4	45	50%	−35	50%	75	50%	−65	50%	0
5	40	50%	−35	50%	75	50%	−50	50%	−10
6	40	50%	−35	50%	75	50%	−45	50%	−12.5
7	35	50%	−35	50%	75	50%	−40	50%	−17.5

Note: EV_A_ and EV_B_ are the expected values of option A and option B, respectively.

**Table 3:** Demographics of the survey sample and census population statistics.

Variable	Description	Mean	Std. Dev.	Provincial Average
Gender	Male = 1, female = 0	0.49	0.50	0.51
Age	Age of the respondent	59	12.45	n/a
Labor	Numbers of labors (including oneself)	3.14	1.36	n/a
Education	Years of education	5.60	4.03	n/a
Hhsize	Household size	5.51	1.81	3.03
Landowned	Farm size (hectare)	0.22	0.22	n/a
Income	Total household income (USD/month)	610.78	2961.89	653.60

**Table 4:** Statistical table of risk cognition of farmers’ adaptation to climate change.

Climate Change Cognition	Strongly Agree	Agree	General	Disagree	Strongly Disagree
Climate change has seriously affected your life	17.10%	51.75%	23.25%	7.89%	0
The possibility of further climate change in the future will be very high	10.53%	51.75%	33.33%	3.51%	0.88%
Actions can mitigate the effects of climate change	0.44%	7.46%	42.54%	46.05%	3.51%
My ability to deal with climate change is very high	0.44%	7.46%	50.44%	31.14%	10.53%
The cost of taking climate change adaptation measures is low	0.88%	3.95%	19.30%	56.14%	19.74%

**Table 5:** Statistical description of farmer’s switching point in the experiment.

Risk-Aversion Experiment			Loss-Aversion Experiment		
Switching Point	Frequency	Percentage (%)	Switching Point	Frequency	Percentage (%)
2	15	6.58	Always B	26	11.40
3	11	4.82	2	9	3.95
4	21	9.21	3	15	6.58
5	40	17.54	4	15	6.58
6	30	13.16	5	58	25.44
7	33	14.47	6	30	13.16
8	14	6.14	7	18	7.89
Always A	55	24.12	Always A	46	20.18
Cross choice	9	3.95	Cross choice	11	4.82

Note: there is no all-B situation in the risk-aversion experiment. Task 1 of this experiment is to test whether participants understood the experiment or not.

**Table 6:** Farmers’ adaptation measures taken to cope with climate change.

Adaptation to Climate Change Decisions	Sample Size of the Measure Taken ^a^	Percentage
Planting new seed varieties	166	72.81%
Adjusting pesticide application behavior	148	64.91%
Adjusting fertilization behavior	136	59.65%
Improving irrigation method/frequency	97	42.54%
Diversified planting	87	38.16%

Note: ^a^ the adaption measures taken are multiple choices.

## Appendix A

**Table A1:** Main structure of the survey questionnaire.

Question Categories	Questionnaire
1. Farmers’ climate change adaptive behavior	In order to cope with climate change, have you taken any adaptation measures? □ Plant new seed varieties □ Adjust pesticide application □ Adjust fertilizer application □ Improve Irrigation methods/frequency □ Diversify planting
2. Farmers’ risk cognition	Please read the following statements and tell us your opinion on a scale of 5 (strongly agree) to 1 (strongly disagree). □ Climate change has severely affected your daily life □ The possibility of climate change in the future will be very high □ Taking adaptation actions can mitigate the effects of climate change □ My ability to deal with climate change is very high □ The cost of climate change adaptation measures is low
3. Respondents’ background information	How old are you? What is your highest level of education? What is your average monthly household income?
